# Long-wavelength room-temperature luminescence from InAs/GaAs quantum dots with an optimized GaAsSbN capping layer

**DOI:** 10.1186/1556-276X-9-36

**Published:** 2014-01-17

**Authors:** Antonio D Utrilla, Jose M Ulloa, Alvaro Guzman, Adrian Hierro

**Affiliations:** 1 Institute for Systems based on Optoelectronics and Microtechnology (ISOM) and Departamento de Ingeniería Electrónica, Universidad Politecnica de Madrid, Ciudad Universitaria s/n, Madrid 28040, Spain

**Keywords:** InAs, Quantum dots, GaAsSbN, Capping layer, Growth rate, Luminescence

## Abstract

An extensive study on molecular beam epitaxy growth conditions of quaternary GaAsSbN as a capping layer (CL) for InAs/GaAs quantum dots (QD) was carried out. In particular, CL thickness, growth temperature, and growth rate were optimized. Problems related to the simultaneous presence of Sb and N, responsible for a significant degradation of photoluminescence (PL), are thereby solved allowing the achievement of room-temperature (RT) emission. A particularly strong improvement on the PL is obtained when the growth rate of the CL is increased. This is likely due to an improvement in the structural quality of the quaternary alloy that resulted from reduced strain and composition inhomogeneities. Nevertheless, a significant reduction of Sb and N incorporation was found when the growth rate was increased. Indeed, the incorporation of N is intrinsically limited to a maximum value of approximately 1.6% when the growth rate is at 2.0 ML s^−1^. Therefore, achieving RT emission and extending it somewhat beyond 1.3 μm were possible by means of a compromise among the growth conditions. This opens the possibility of exploiting the versatility on band structure engineering offered by this QD-CL structure in devices working at RT.

**PACS:**

81.15.Hi (molecular beam epitaxy); 78.55.Cr (III-V semiconductors); 73.21.La (quantum dots)

## Background

Tailoring the band structure and optical properties of the technologically mastered InAs/GaAs quantum dots (QDs) has been the focus of many efforts in the last decade. The use of a GaAsSb strain-reducing capping layer (CL) has been widely studied for that purpose [1-4]. The presence of Sb raises the valence band (VB) of GaAs [5] allowing the extending of QD emission over a wide wavelength range. Moreover, Sb suppresses the decomposition of GaAs-capped QDs [6] and has been shown to provide devices with improved characteristics [7-10]. Within this approach, the rise of the VB induced by the presence of Sb makes the band alignment structure become type II for contents of Sb above structures 14% to 16% [2-4]. A further step forward which has been recently proposed is the addition of N to the ternary GaAsSb CL. The incorporation of N in GaAs, according to the band anticrossing model [11], reduces only the conduction band (CB) of GaAs the same way Sb raises only its VB. Therefore, the use of the quaternary GaAsSbN CL on InAs/GaAs QDs allows tuning independently the electron and hole confinement potentials, as it has already been demonstrated [12]. Moreover, this approach allows modifying the band alignment from type I to type II in both the CB and the VB. Thus, the versatility in band structure engineering makes this system a promising candidate for optoelectronic device applications of InAs/GaAs QDs requiring different band alignments. For instance, type-II InAs/GaAs QDs with a larger carrier lifetime could enhance the carrier extraction efficiency in photodetectors or QD solar cells, as proposed for the GaSb/GaAs system [13]. Moreover, the strongly improved responsivity recently demonstrated in GaAsSb-capped InAs/GaAs QD infrared photodetectors (QDIPs) [8] could be spectrally tuned by controlling the N content in the quaternary CL. Light-emitting devices, such as laser diodes (LD), could also benefit from this approach. As an example, the difficulty to achieve long lasing wavelengths in GaAsSb-capped InAs/GaAs QD LDs due to the inhibition of type-II transitions [10] could be overcome by adding N, which allows reaching longer wavelengths while keeping the type-I band alignment.

However, significant structural changes of the capping layer due to the addition of N have been found to take place [14]. Strain and compositional inhomogeneities are induced during the CL growth, yielding a degradation of the luminescence such that, as far as we know, no room-temperature (RT) emission has been reported to date using such a CL. Nevertheless, the resulting morphology of the CL could be modified through the growth conditions. Growth parameters such as growth temperature or growth rate could significantly influence the mass transport phenomena and composition modulation. Therefore, a need arises to find the optimal growth conditions in order to exploit the promising properties of this QD-CL system in optoelectronic applications. In this work, we study the effect of modifying the CL growth temperature, thickness, and growth rate on QD luminescence. RT photoluminescence (PL) is shown to be achievable through different growth conditions, and extending the emission to 1.3 μm is possible by means of the appropriate combination of the growth parameters.

## Methods

All of the analyzed samples were grown by solid source molecular beam epitaxy on *n*^+^-doped GaAs (001) substrates. The QD layers were always grown under the same conditions by depositing 2.8 monolayers (ML) of InAs at 450°C and 0.04 ML s^−1^ on an intrinsic 0.5-μm-thick GaAs buffer layer. The GaAsSbN CL was grown under the reference conditions discussed below, modifying only one of the growth parameters for each series of samples. A 250-nm-thick GaAs layer was grown on top of the GaAsSbN capping. Sb was supplied from an effusion cell, while active N was generated from a radio-frequency (RF) plasma source with a 0.1-sccmflow of pure N_2_. The samples were characterized by PL measurements at 15 K and RT. A He-Ne laser was used as the excitation source, and low-temperature (LT) measurements were done using a closed-cycle He cryostat. The emitted light from the samples was dispersed by a 1-m spectrometer and detected with a liquid nitrogen-cooled Ge detector through standard lock-in techniques.

## Results and discussion

First, it is necessary to establish the reference growth conditions for the GaAsSbN CL as a starting point from which one of the parameters will be modified in each series of samples. Thus, as reference conditions for the CL growth, those used in previous studies are considered [12], i.e., a 470°C growth temperature, a ratio of As_4_/Ga beam equivalent pressure of 32, a thickness of 5 nm, and a growth rate of 1 ML s^−1^. Regarding the N and Sb contents, a power of 140 W for the RF plasma source and a temperature of 335°C for the Sb effusion cell were chosen as reference source conditions. These conditions correspond in our system to nominal contents of 2.5% of N and 15% of Sb. In order to support the accuracy of these values, three samples consisting of single GaAsSb, GaAsN, and GaAsSbN quantum wells (QWs) were grown under the same reference conditions. For the GaAsSb QW sample, an emission peak of 1.242 eV at RT was found, corresponding to an Sb content of approximately 15% according to theoretical and experimental results for such a GaAsSb QW thickness [15]. Regarding the GaAsN QW, a content of N around 2.3% can be estimated when comparing with similar reported QWs [16]. The LT PL from the quaternary QW sample shifted from the GaAs gap energy a higher value (527 meV) than the addition of shifts in the GaAsSb (216 meV) and GaAsN (255 meV) QW samples. This is in agreement with studies reporting a facilitated incorporation of N by the presence of Sb [17,18]. Indeed, the difference of 56 mV points to a higher N content corresponding to approximately 2.8%. For these N and Sb contents, the system will still be in the type-I band alignment region [12]. Furthermore, since the Sb/N ratio is larger than 2.6 (the condition for lattice matching to GaAs) it can be assumed that the GaAsSbN layer grows under compressive strain on GaAs and will act as a strain-reducing CL.

### Capping layer growth temperature

**Figure 1:**
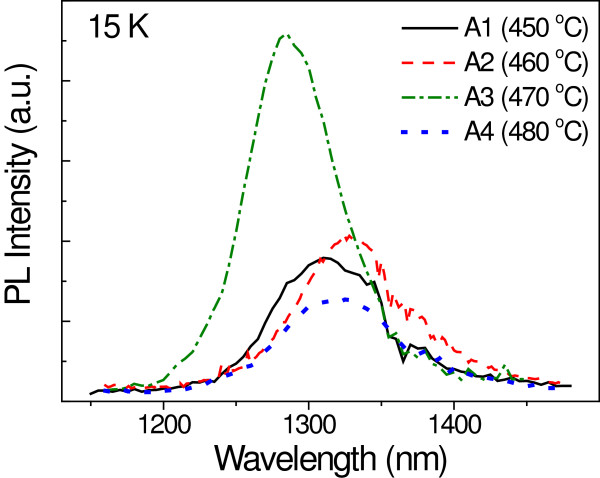
PL spectra at 15 K as a function of the CL growth temperature.

First, the study focuses on finding the optimal growth temperature for the GaAsSbN CL. The incorporation of N in GaAs has been found to be temperature independent in a wide range of temperatures from 400°C to 480°C [19] or even higher temperatures [20,21]. However, for temperatures higher than that, N incorporation is strongly reduced. This is probably induced by the temperature-enhanced desorption of N from the growth surface, as it has been theoretically predicted [22]. On the other hand, as expected from the fact that Sb has a higher sublimation energy than As [23], increasing the temperature affects substantially the incorporation of Sb [24,25]. Thus, Sb desorption has been found to increase with temperature, becoming substantial above 490°C [24]. Hence, in order to avoid a significant desorption of both Sb and N as well as a substantial modification of the InAs QDs, we studied the effect of the CL growth temperature in a range between 450°C and 480°C. A series of four samples was grown with CL growth temperatures set to 450°C, 460°C, 470°C, and 480°C (labeled as A1, A2, A3, and A4, respectively). Figure 1 shows the PL spectra of the four samples. The small peak wavelength shifts observed do not follow any tendency with the growth temperature and are likely within the reproducibility error bar. Nevertheless, an improvement of the luminescence properties can be observed with increasing the growth temperature from 450°C up to 470°C, being more remarkable for the last temperature case. The full width at half maximum (FWHM) is slightly reduced, and the integrated intensity is approximately doubled when raising the temperature within this range. However, above 470°C, the integrated PL intensity is reduced by approximately 65% and the FWHM is slightly increased. According to these results, the CL growth temperature is set to 470°C for the remaining analysis.

### Capping layer thickness

**Figure 2:**
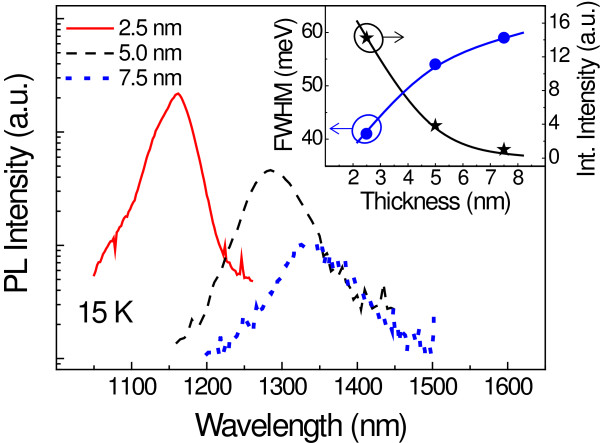
**PL spectra at 15 K for samples with different CL thicknesses.** The inset shows the FWHM and the integrated intensity as a function of the CL thickness. Lines are guides to the eye.

In order to analyze the impact of the CL thickness on the PL properties, a series of samples with 2.5-, 5.0-, and 7.5-nm-thick GaAsSbN CLs was grown (labeled as B1, B2, and B3, respectively). Figure 2 shows the PL spectra at 15 K of the three samples, and the extracted FWHM and integrated intensity are represented in the inset. Reducing the CL thickness from 7.5 to 2.5 nm induces a considerable blueshift, leading also to a decrease of 20 meV in the FWHM and to a significant enhancement in the integrated intensity by a factor of 15. Thus, a clear tendency of the luminescence properties with the CL thickness can be observed, whereby the peak wavelength is red-shifted as the CL thickness increases, accompanied by a significant degradation of the radiative efficiency. This redshift could arise from several mechanisms. First, a thicker strain-reducing CL should induce a reduction of the compressive strain inside the QD. Second, and as it happens in GaAsSb-capped QDs [26], the QD size may be larger for thicker GaAsSbN CLs. The degradation of the radiative efficiency likely originated from a higher composition modulation. Indeed, a higher composition modulation is expected for thicker CLs since they accumulate a larger amount of strain, yielding a more pronounced interface roughness. This clustering and roughness would directly impact the carrier injection efficiency into the InAs QDs, decreasing the radiative efficiency of the PL.

### Capping layer growth rate

#### The GaAsSbN CL

**Figure 3:**
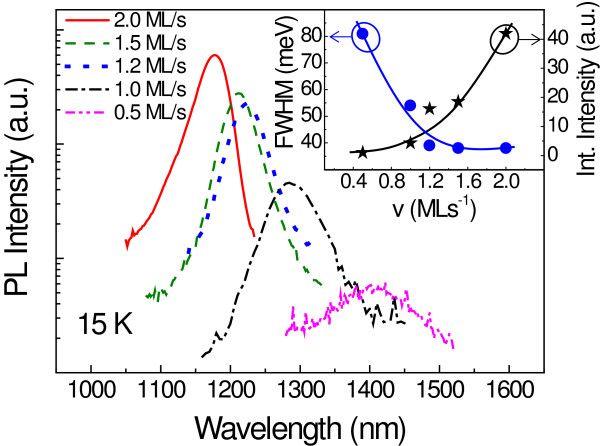
**PL spectra at 15 K for samples with different CL growth rates.** The inset shows the FWHM and the integrated intensity as a function of the CL growth rate. Lines are guides to the eye.

A series of samples was grown wherein the only modified parameter was the growth rate of the quaternary GaAsSbN CL while the rest of the growth parameters were kept at their reference values. Five samples with CL growth rates of 0.5, 1.0, 1.2, 1.5, and 2.0 ML s^−1^ were grown (labeled as C1, C2, C3, C4, and C5, respectively). Figure 3 shows the PL spectra for this series of samples with their integrated intensity and FWHM evolution depicted in the inset. A significant enhancement of the PL properties with the growth rate is observed. The integrated intensity is improved up to 40 times when going from 0.5 to 2.0 ML s^−1^, and the FWHM is reduced to 38 meV for rates above 1.2 ML s^−1^. Moreover, samples with the CL grown at and above 1.2 ML s^−1^ showed RT luminescence (the RT PL results will be discussed below). However, the emission is blue-shifted when the growth rate is increased, which suggests a reduced N and/or Sb incorporation in the CL.

#### The GaAsSb and GaAsN CLs

**Figure 4:**
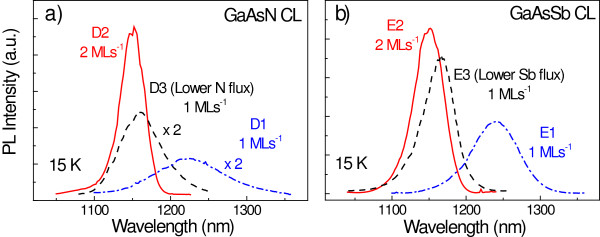
**PL spectra at 15 K of ternary CL samples as a function of the growth rate. (a)** Spectra of samples containing a GaAsN CL grown at 1 and 2 ML s^−1^ (D1 and D2, respectively), together with that of a sample with the CL grown at 1 ML s^−1^ using a lower RF plasma source power (D3). **(b)** Spectra of samples containing a GaAsSb CL grown at 1 and 2 ML s^−1^ (E1 and E2, respectively), together with that of a sample with the CL grown at 1 ML s^−1^ using a lower Sb effusion cell temperature (E3).

In order to clarify the effect of the growth rate on the N and Sb contents individually, samples either with a GaAsN (samples D1 and D2) or a GaAsSb (samples E1 and E2) CL were also studied. The CL growth rates were 1 and 2 ML s^−1^ for D1/E1 and D2/E2, respectively. All these samples were grown under the same conditions as the quaternary counterpart. Figure 4a shows the PL spectra for the GaAsN CL grown at 1 ML s^−1^ (dashed-dotted blue line) and 2 ML s^−1^ (continuous red line). A blueshift together with a strong PL improvement can be also appreciated here when the growth rate of the CL is increased, as it happens for the case of the quaternary. Since the N incorporation was found to be inversely proportional to the growth rate [19,21], the blueshift can be attributed to a reduced N content. Thus, the sample with the CL grown at 2 ML s^−1^ has a lower N concentration than the 1-ML s^−1^ CL sample.

In order to decouple the effect of the N concentration on the PL properties from that of the growth rate, a third sample was grown at 1 ML s^−1^ (D3, dashed black line in Figure 4a). The N RF plasma power was decreased until the PL peak energy matched that of D2, i.e., until the N concentration was the same. A comparison of the PL from samples D2 and D3 (equal N concentration and 2/1-ML s^−1^ growth rates, respectively) now clearly shows that the PL improvement at higher growth rates is not only due to a reduced N incorporation but also due to an improved structural quality of the CL.

In the case of the GaAsSb CL, a blueshift and a moderate PL enhancement is observed with increasing growth rate (Figure 4b), also indicative of a lower Sb incorporation. This behavior contradicts that reported for GaAsSb QWs grown at growth rates below 1 ML s^−1^[24], but no reports for higher growth rates are available in the literature. Like in the case of the GaAsN CL, a third sample was grown to decouple the effect of the growth rate and the Sb concentration. This sample (E3, dashed black line in Figure 4b) had a lower Sb content to match that of E2 (similar PL peak energy) and a 1-ML s^−1^ CL growth rate. Contrary to the case of GaAsN, increasing the growth rate while maintaining the Sb content constant seems to produce a minimum improvement of the PL (see the PLs from E2 and E3 in Figure 4b). Thus, we can conclude the sole increase of the growth rate (samples E1 and E2) leads to a decreased Sb content that is entirely responsible for the improved PL. Indeed, it has been shown that the PL of GaAsSb-capped InAs QDs is degraded for Sb contents above 12% [27], so reducing the initial content (approximately 15%) should result in an improved PL.

### Quantification of the Sb/N content reduction

**Figure 5:**
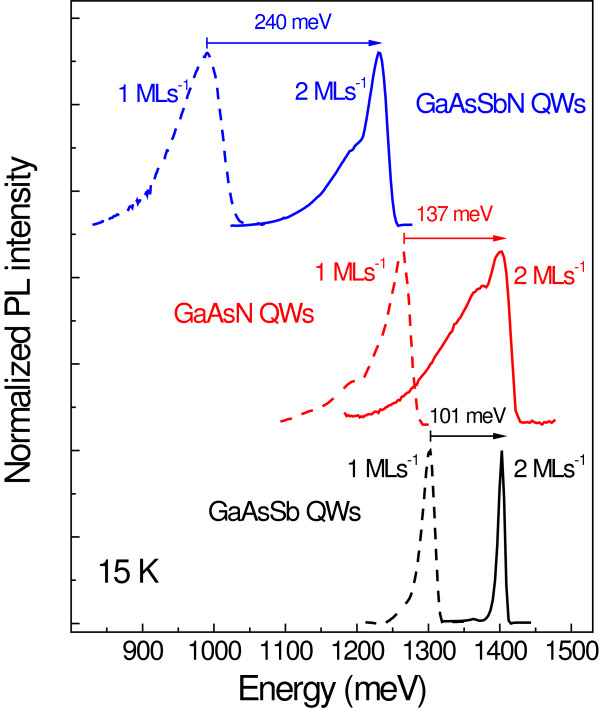
**PL spectra at 15 K for GaAsSb, GaAsN, and GaAsSbN QWs grown at 1 and 2 ML s**^**−1**^**.** The spectra corresponding to different materials are shifted in the vertical axis for the sake of clarity. Arrows indicate the respective blueshifts induced by the increased growth rate.

In order to determine the reduction of the Sb and N contents when growing the CL at the highest rate, samples consisting of single GaAsSb, GaAsN, and GaAsSbN QWs were grown at 1 and 2 ML s^−1^ using the reference source conditions. Figure 5 shows the PL spectra from these samples, where PLs from samples grown at 1 ML s^−1^ appear as dashed lines while those from samples grown at 2 ML s^−1^ are represented by continuous lines. Regarding the GaAsSb QWs (black lines), the increase in growth rate induces a blueshift of 101 meV, from which a significant reduction of the Sb content of approximately 8% can be deduced [15]. Likewise, the emission from GaAsN QW (red lines) is also strongly blue-shifted as a consequence of the reduced N incorporation. From the blueshift of 137 meV found for this case, a reduction of N content of approximately 1.2% is estimated [16]. The N content is therefore reduced to about half when doubling the growth rate, which is in good agreement with what is expected from the inverse linear N incorporation dependence on the growth rate [19,21]. In the case of the GaAsSbN QW (blue lines), the observed shift is 240 meV, which corresponds very well to the addition of the shift values for the two ternaries, indicating a similar decrease of Sb and N of 8% and 1.2%, respectively. Therefore, Sb and N contents of 7% and 1.6% are expected for the GaAsSbN CL grown at 2 ML s^−1^.

### Comparison among the three CL materials

**Figure 6:**
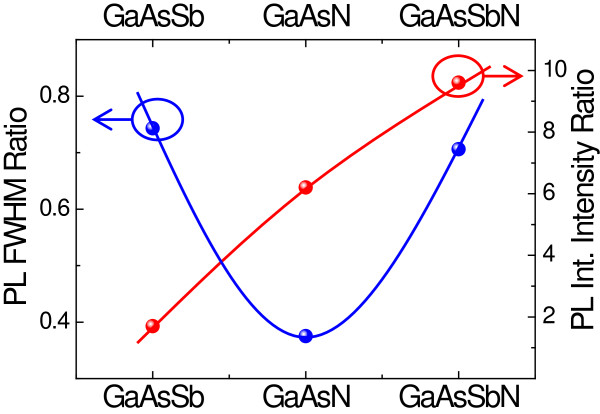
**Growth rate-induced improvements in the PL spectra for the three CL materials.** FWHM and the integrated intensity ratios between 2- and 1-ML s^−1^ grown samples for GaAsSb, GaAsN, and GaAsSbN CLs.

Figure 6 shows PL FWHM and integrated intensity ratio between the QD samples grown at 2 and 1 ML s^−1^ for the three cases, the ternaries GaAsSb and GaAsN, and the quaternary CL samples. A reduction of the FWHM of 65% is found for the GaAsN CL sample, stronger than the 25% to 30% observed for the GaAsSb and GaAsSbN CL samples. On the other hand, the integrated intensity significantly increases for the GaAsN and the GaAsSbN CL samples by a factor of 6.2 and 9.6, respectively. These results show that increasing the growth rate has a particularly strong positive impact in N-containing structures. This could be related to a reduced composition modulation that resulted from a lower diffusion of N and Sb atoms on the growth surface. In particular, the reduced FWHM of the PL seems to indicate a homogenization of the CL composition on top of the QDs, where a strong Sb accumulation induced by the presence of N was reported when growing at 1 ML s^−1^[14]. Moreover, the shorter time of exposure to the plasma could be attenuating the damage induced by the excited N species, mainly N ions, coming from the plasma source. This would result in a reduced defect density.

### Extending the emission wavelength

**Figure 7:**
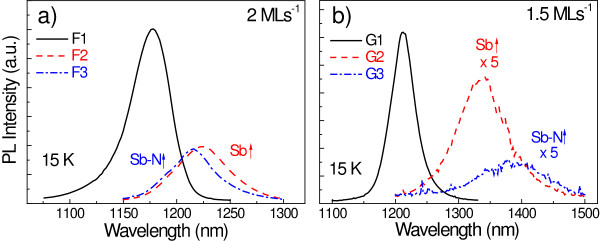
**PL spectra at 15 K for samples with different Sb and N contents.** PL spectra when increasing the flux of Sb and N during the growth of the CL at **(a)** 2.0 ML s^−1^ and **(b)** 1.5 ML s^−1^.

Our goal is to extend the emission wavelength through the best growth conditions found from the different approaches analyzed above. Since the most significant improvement was found when the growth rate of the CL is increased, the efforts will first focus on trying to extend the emission by adding higher amounts of Sb and N in the CL grown at 2 ML s^−1^. The reference values will be used for the other parameters. Three samples with the CL layer grown at 2 ML s^−1^ were studied: the first one with the reference parameters for N and Sb sources (sample F1), the second one by raising the Sb effusion cell temperature to 345°C (sample F2), and the last one by increasing both the Sb cell temperature to 345°C and the RF plasma source power to 210 W (sample F3). The PL spectra from this series of samples are shown in Figure 7a. It can be observed that increasing the Sb content in the CL leads to a red-shifted emission peak with a simultaneously weakened luminescence. However, it was impossible to incorporate a higher N content at this growth rate, finding a similar spectrum for sample F3 as that of sample F2, with no significant peak shift. This means that the additional active N provided is not being incorporated substitutionally into the lattice.

A similar study was carried out also for a lower growth rate of 1.5 ML s^−1^. The three samples described in the previous paragraph, with the same parameters for the Sb and N sources, were reproduced with a CL growth rate of 1.5 ML s^−1^ (G1, G2 and G3, respectively). The PL spectra are shown in Figure 7b. The PL peak redshift in sample G2 is now 97 meV, as compared to 40 meV at 2 ML s^−1^. This means that a higher amount of Sb is now incorporated for the same Sb flux than at 2 ML s^−1^. Moreover, adding higher N contents is still possible at this lower growth rate, resulting in a long wavelength peak close to 1.4 μm at 15 K (sample G3).

This result shows that a strict limitation exists related to N incorporation in the GaAsSbN CL at high growth rates. N contents above approximately 1.6% cannot be incorporated into the lattice when growing at 2 ML s^−1^. This forces us to limit ourselves to lower growth rates in order to achieve long emission wavelengths.

### Results at RT

**Figure 8:**
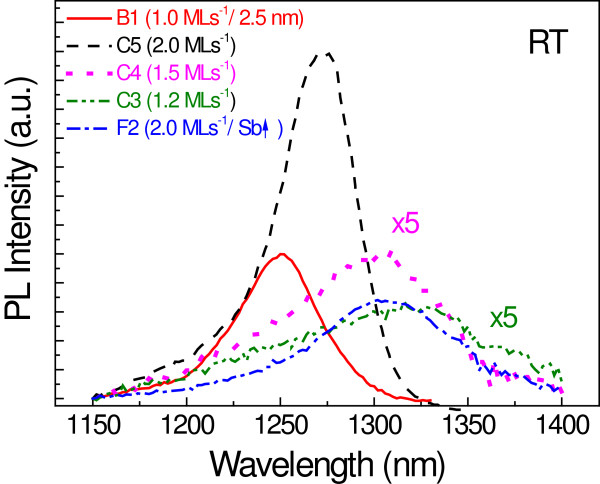
RT PL spectra for samples emitting around 1.3 μm.

Figure 8 shows the RT PL spectra for all the samples from this paper emitting near 1.3 μm. As it can be observed, RT emission was obtained through different approaches. When using a the 2.5-nm-thick GaAsSbN CL (sample B1), RT emission at 1,250 nm was obtained. On the other hand, as the LT results suggested, a more intense RT emission can be achieved when increasing the growth rate of the CL. Sample C5 (2 ML s^−1^) emits at 1,270 nm with improved luminescence properties, showing an integrated intensity more than twice larger than that of sample B1, together with a PL line width of only 39 meV. Longer wavelengths were achieved from samples with the CL grown at 1.5 ML s^−1^ (C4) and 1.2 ML s^−1^ (C3), emitting at 1,307 and 1,329 nm, respectively, but with a more deteriorated luminescence as the growth rate is reduced. By adding a higher Sb content to the CL grown at 2 ML s^−1^, it is also possible to reach peak wavelengths somewhat beyond 1.3 μm. Indeed, sample F2 emits at 1,308 nm, showing a significantly more intense luminescence than samples C3 and C4 with a narrower FWHM, which was hardly widened when the temperature was increased from 15 K up to RT. This again points to the benefits provided by the highest growth rate, which allows achieving long emission wavelengths with improved luminescence properties. The obtained results represent the first step towards using GaAsSbN CLs in RT device applications.

## Conclusions

The effect of modifying the growth conditions of the quaternary GaAsSbN CL on the PL properties of the InAs/GaAs QDs has been analyzed. Regarding growth temperature, 470°C was found to be the optimum value. A clear tendency was found when the CL thickness was modified, whereby the peak is red-shifted and the PL is degraded as the CL thickness increased. The best results were found when the CL growth rate was increased. The strong PL improvement at high growth rates up to 2 ML s^−1^ is shown to be specific for N-containing structures and likely related to a reduced composition modulation and plasma ion-induced defect density. Nevertheless, a strict limitation regarding N incorporation is found when the CL is grown at 2 ML s^−1^, which forces one to remain at lower values in order to reach longer wavelengths. RT PL is obtained through different growth conditions, some of them leading to 1.3-μm emission. The best luminescence properties were found for the highest CL growth rate, being still possible to extend the emission wavelength by adding higher Sb contents. The obtained outcomes from the growth optimization of this system could represent a starting point from which the versatility of the GaAsSbN CL might be exploited for real device applications.

## Competing interests

The authors declare that they have no competing interests.

## Authors’ contributions

ADU and JMU designed the samples and the experiments. ADU grew the samples and did the photoluminescence measurements under the supervision of JMU. AG and AH helped in discussing the results and in preparing the manuscript. All authors read and approved the final manuscript.
